# GLMB Tracker with Partial Smoothing

**DOI:** 10.3390/s19204419

**Published:** 2019-10-12

**Authors:** Tran Thien Dat Nguyen, Du Yong Kim

**Affiliations:** 1 School of Electrical Engineering, Computing and Mathematical Sciences, Curtin University, Bentley 6102, Australia; 2 School of Engineering, RMIT University, Melbourne 3000, Australia

**Keywords:** labeled RFS, RTS smoother, GLMB filter

## Abstract

In this paper, we introduce a tracking algorithm based on labeled Random Finite Sets (RFS) and Rauch–Tung–Striebel (RTS) smoother via a Generalized Labeled Multi-Bernoulli (GLMB) multi-scan estimator to track multiple objects in a wide range of tracking scenarios. In the forward filtering stage, we use the GLMB filter to generate a set of labels and the association history between labels and the measurements. In the trajectory-estimating stage, we apply a track management strategy to eliminate tracks with short lifespan compared to a threshold value. Subsequently, we apply the information of trajectories captured from the forward GLMB filtering stage to carry out standard forward filtering and RTS backward smoothing on each estimated trajectory. For the experiment, we implement the tracker with standard GLMB filter, the hybrid track-before-detect (TBD) GLMB filter, and the GLMB filter with objects spawning. The results show improvements in tracking performance for all implemented trackers given negligible extra computational effort compared to standard GLMB filters.

## 1. Introduction

While single-object tracking algorithms have been studied extensively for more than half a century, multi-object tracking is currently a trending topic in signal processing society due to its extensive applications. The challenges of the multi-object tracking problem arise in the context of miss-detection, false alarms, object thinning, and appearing processes. To tackle these problems, several frameworks have been put forward in the literature such as the Joint Probabilistic Data Association (JPDA) [1], multiple hypotheses tracking [2], and recently, Random Finite Sets (RFS) [2]. In particular, RFS forms the mathematical basis of many modern multi-object filters such as Probability Hypothesis Density (PHD) filter [3,4,5,6,7], cardinalized PHD (CPHD) filter [8,9,10], multi-Bernoulli filter [11,12], the Generalized Labeled Multi-Bernoulli (GLMB) filter [13,14,15,16,17,18,19], and its approximation the Labeled Multi-Bernoulli (LMB) filter [20,21]. In many applications, tracking algorithms rely on the standard point measurements to update the object states; in contrast, TBD [22,23,24,25] is an alternative approach that bypasses the detection module to directly exploit the observed spatial data. This technique is introduced under the RFS framework in Reference [26] with the development of the so-called separable likelihood model and, recently, in a hybrid (combination of standard observation and separable observation models) approach in Reference [27]. In terms of system modelling, in many multi-object tracking scenarios, it is sufficient to consider object thinning and appearing processes via survivals, deaths, and instantaneous birth models. However, in many practical applications, new objects are also generated from a set or a subset of existing objects. In the context of RFS-based filtering techniques, such spawning models have been proposed for CPHD filter in References [28,29] and for GLMB filter in Reference [30]. Because these spawning models correctly reflect the physical state of the systems with spawning objects, the accuracy of the estimate is improved.

The early works on practical smoothing algorithms for single-object tracking were introduced by Bryson and Frazier [31]; by Rauch, Tung, and Striebel [32]; and subsequently by Fraser and Potter [33]. Later on, many alternative smoothing algorithms for a nonlinear dynamic model were proposed in References [34,35,36,37,38,39]. Recently, the closed-form solution for the Gaussian Mixture (GM) forward-backward smoother was derived in Reference [3]. Furthermore, the smoother for the multi-sensor tracking problem is addressed in Reference [40], while the smoothing solution for maneuvering-object tracking is presented in Reference [41]. In Reference [42], a method for joint tracking smoothing of object trajectory based on function fitting is also proposed. In a multi-object tracking context, several smoothing techniques have been put forward in the literature despite the challenge of the large smoothing state space. In particular, smoothing for PHD filter is introduced in References [43,44,45], while smoothing for CPHD filter and Multi-Bernoulli filter are given respectively in References [46,47]. Multiple objects can also be tracked with a fixed-lag smoother via Interacting Multiple Model (IMM) in Reference [48]. Closed-form solution for forward-backward smoothing based on GLMB RFS is introduced in Reference [49], and recently, multi-scan smoothing technique was proposed in Reference [50] with an efficient implementation based on Gibbs sampling, which can easily handle 100 scans. This is an unprecedented advance over traditional multi-scan solutions, which can only handle about 10 scans.

The labeled RFS approach has several theoretical and practical advantages over unlabeled approaches. The first is that labeled RFS filters can provide trajectory estimates naturally without heuristics, whereas this is not possible with unlabeled RFS filters; see Reference [51]. The second is that labeled RFS can provide ancestry information in a principled manner, whereas unlabeled RFS does not have the mechanism to do this (even with smoothing) [30]. The third is that labeled RFS admits analytical solutions such as the GLMB densities that are still valid RFS densities after any truncation, whereas unlabeled RFS cannot; see [51]. The fourth is that the truncation error (or error bound) for labeled RFS, such as GLMB, is available analytically, whereas this is not available for unlabeled RFS [13]. Consequently, truncation-based unlabeled RFS algorithms are heuristics [51]. Numerically, labeled RFS filters such as the GLMB have been demonstrated to be scalable in the number of objects [52], number of scans [50], and number of sensors [53]. Hence, the GLMB is a versatile class of models for multi-sensor multi-object problems.

In this paper, we introduce a tracker based on the GLMB filters and a modification of the multi-scan estimator proposed in Reference [50]. After the forward GLMB filtering stage, a pre-smooth stage is implemented to eliminate short-term tracks, which are usually initiated by false births or spawns. The threshold to prune these tracks varies depending on the tracking scenario. Subsequently, a multi-scan estimator which consists of a standard single-object filter and an Rauch–Tung–Striebel (RTS) smoother is applied on each estimated trajectory to produce smoothed estimates. As the proposed multi-scan estimator operates only on the estimated trajectories, the complexity is much lower than the full smoothing solution proposed in Reference [50]. Especially, this proposed tracker can completely eliminate track fragmentation as the multi-scan estimator estimates the entire trajectories but not single-scan multi-object states as in standard GLMB filters [13,15,16]. We demonstrate the application of the proposed tracker on both a standard measurement model and a TBD measurement model as well as tracking scenario with object spawning.

The structure of this paper is as follows. In Section 2, we provide background information on labeled RFSs, the multi-object transition kernel, the observation models, and the single-object RTS smoother for linear and nonlinear dynamic models. In Section 3, we propose the tracker based on the GLMB filters and the multi-scan estimator. In Section 4, we first show the experimental results for tracking with the standard observation model in linear and nonlinear tracking scenarios. We then show the tracking results of the proposed algorithm with a hybrid observation model. Finally, we demonstrate the performance of the algorithm on tracking biological cells in an image sequence where spawning process occurs.

## 2. Background

### 2.1. The Labeled RFS

Throughout this article, we adhere to the following notations. The set exponential is denoted as ${\left[h\left(\cdot \right)\right]}^{X}=\prod_{x\in X}h\left(x\right)$ while the inner product notation is denoted as 〈f,g〉=∫f(x)g(x)dx. The generalization of the Kronecker delta is denoted as follows:

$$\delta_{Y}\left(X\right)=\left\{\begin{array}{cc} 1 & X=Y\\ 0 & X\neq Y \end{array}\right.$$

The set inclusion function is written as follows:

$$1_{Y}\left(X\right)=\left\{\begin{array}{cc} 1 & X\subseteq \;Y\\ 0 & otherwise \end{array}\right.$$

X denotes the labeled set of objects, while x=(x,l) denotes a single labeled object, specifically, x∈X and l∈L, where X and L are respectively the kinematic state space and the discrete labels space at the current time step. L is a label extraction function, i.e., L(x)=l and F(X) denote sets of finite subsets of X. The “+” sign is used to indicate the next time step when applicable.

The Finite-Set Statistics (FISST) integration is defined as follows [54]:

$$f\left(X\right)\delta X=\sum_{i=0}^{\infty }\frac{1}{i!}\int_{X^{i}}f\left(\left\{x_{1},\ldots ,x_{i}\right\}\right)d\left(x_{1},\ldots ,x_{i}\right)$$

In multi-object tracking problem, the cardinality of object sets varies when objects enter or leave the surveillance region. As RFS is a random set of points in the sense that the number of points in the set is random and the points themselves are also random and unordered [54], a set of random objects can be naturally characterized as a RFS. Being introduced systematically for the first time in Reference [13], the labeled RFS incorporates the identities of elements into the unlabeled counterpart. Precisely, with the state space X and marks space L, the labeled RFS is a marked simple point process whereas each realization has a distinct label [13,15]. The distinct label property is satisfied when X has the same cardinality as its labels L(X). Given this, the distinct label indicator can be written as follows [16]:

(1) $$\begin{array}{c} \Delta \left(X\right)=\delta_{\left|X\right|}\left(|L\left(X\right)|\right) \end{array}$$

The introduction of labeled RFS to the multi-object tracking problem allows direct estimation of trajectories which cannot be done previously with conventional RFS without a separate labeling scheme.

### 2.2. The Multi-Object Transition Kernel

In standard tracking scenario, an existing object can either survive or die in the next time step. The surviving objects are modeled as an LMB RFS with a survival probability of $p_{S}\left(x,l\right)$, a disappearance probability of $q_{S}\left(x,l\right)=1-p_{S}\left(x,l\right)$, and a spatial distribution of $f_{S+}\left(x_{+}|x,l\right)$. The model for such surviving objects is given as follows [13,15,16]:

(2) $$\begin{array}{cc} f_{S+}\left(X_{S+}|X\right) & =\Delta \left(X\right)\Delta \left(X_{S+}\right)1_{L(X)}\left(L\left(X_{S+}\right)\right){\left[\Phi_{S+}\left(X_{S+}|\cdot \right)\right]}^{X} \end{array}$$

where

$$\begin{array}{cc} \Phi_{S+}\left(X_{S+}|x,l\right) & =\sum_{\left(x_{+},l_{+}\right)\in X_{S+}}\delta_{l}\left(l_{+}\right)p_{S}\left(x,l\right)f_{S+}\left(x_{+}|x,l\right)+\left[1-1_{L(X_{S+})}\left(l\right)\right]q_{S}\left(x,l\right) \end{array}$$

In addition, the new birth objects can instantaneously appear at each time steps and they are modeled with LMB RFS as follows [13,15,16]:

(3) $$\begin{array}{cc} f_{B+}\left(X_{B+}\right) & =\Delta \left(X_{B+}\right)w_{B}\left(L\left(X_{B+}\right)\right){\left[p_{B+}\right]}^{X_{B+}} \end{array}$$

$$\begin{array}{cc} w_{B}\left(L\left(X_{B+}\right)\right) & =1_{B+}\left(L\left(X_{B+}\right)\right){\left[1-r_{B+}\right]}^{B_{+}-L\left(X_{B+}\right)}{\left[r_{B+}\right]}^{L(X_{B+})} \end{array}$$

Furthermore, in certain scenarios, new objects can also be generated from existing objects, which leads to the need of a spawning model in order to correctly predict the state of the system at the next time step. Recently, a spawning model for GLMB filter has been proposed in Reference [30]; we introduce this model again here as follows for the sake of completeness.

For spawned objects, the naming convention is given as follows: if at time step *k* the label of an object is *l*, then the spawned labels from *l* at the next time step is $l_{spawn}=\left(l,k+1,i\right)$, where *i* is the index to distinguish between different spawned objects from the same parent. Following this convention, the set of all spawned labels in the next time step is $S_{+}=L\times \left\{k+1\right\}\times N$, where N is the set of positive natural numbers [30].

For each spawned object with the label $l_{spawn}\in S_{+}\left(L\left(x\right)\right)$, it will either exist with the probability $p_{T}\left(x;l_{spawn}\right)$ and a spatial distribution $f_{T+}\left(x_{+}|x;l_{spawn}\right)$ or not with the probability $q_{T}\left(x;l_{spawn}\right)=1-p_{T}\left(x;l_{spawn}\right)$.

The density of the set P of new spawned objects from x is formulated as follows [30]:

(4) $$\begin{array}{cc} f_{T+}\left(P|x,l_{spawn}\right) & =\Delta \left(P\right)1_{S_{+}\left(L\left(x\right)\right)}\left(L\left(P\right)\right){\left[\Phi_{T+}\left(P|x;\cdot \right)\right]}^{S_{+}\left(L\left(x\right)\right)} \end{array}$$

where

$$\begin{array}{cc} \Phi_{T+}\left(P|x;l_{spawn}\right) & =\sum_{\left(x_{+},l_{+}\right)}\delta_{l_{spawn}}\left(l_{+}\right)p_{T}\left(x,l_{spawn}\right)f_{T+}\left(x_{+}|x,l_{spawn}\right)+\left[1-1_{L(P)}\left(l_{spawn}\right)\right]q_{T}\left(x,l_{spawn}\right) \end{array}$$

Let Q be a labeled set of objects spawned from X with $L\left(Q\right)\subseteq S_{+}\left(L\left(X\right)\right)$. As all labels sets are disjoint, the FISST convolution theorem [54] can be applied.

(5) $$\begin{array}{cc} f_{T+}\left(Q|X\right) & =\Delta \left(Q\right)1_{S_{+}\left(L\left(X\right)\right)}\left(L\left(Q\right)\right){\left[\Phi_{T+}\left(Q|\cdot \right)\right]}^{X} \end{array}$$

where

$$\begin{array}{cc} \Phi_{T+}\left(Q|x\right) & =[\Phi_{T+}{\left(Q\cap \left(X\times S_{+}\left(L\left(x\right)\right)|x;\cdot \right)\right]}^{S_{+}\left(L\left(x\right)\right)} \end{array}$$

As new birth objects (given in Equation (3)) are independent of the previous time step objects, the overall transition model is given as follows:

(6) $$\begin{array}{cc} f(X_{+}|X) & =f_{S+}\left(X_{S+}|X\right)f_{T+}\left(Q|X\right)f_{B+}\left(X_{B+}\right) \end{array}$$

As the spawned objects depend upon the objects from previous time steps, the prediction step of the filtering stage needs to be done in a joint manner to capture the objects’ dependency. As a result, approximation is needed to convert the joint object distribution to a standard GLMB density for each time step in order to keep the algorithm tractable.

In the scenario where the spawning process is not present, the multi-object transition kernel is reduced to the following:

(7) $$\begin{array}{cc} f(X_{+}|X) & =f_{S+}\left(X_{S+}|X\right)f_{B+}\left(X_{B+}\right) \end{array}$$

### 2.3. The Multi-Object Observation Models

In the RFS multi-object tracking framework, given a set of measurements $Z=\{z_{1:|Z|}\}$, we have a standard observation model of the following form: [54]

(8) $$\begin{array}{cc} g(Z|X) & \propto \sum_{\theta \in \Theta (L(X))}\prod_{(x,l)\in X}\psi_{Z}^{\left(\theta (l)\right)}\left(x,l\right) \end{array}$$

where

$$\begin{array}{cc} \psi_{Z}^{\left(\theta (l)\right)}\left(x,l\right) & =\delta_{0}\left(\theta \left(l\right)\right)q_{D}\left(x,l\right)+\left(1-\delta_{0}\left(\theta \left(l\right)\right)\right)\frac{p_{D}\left(x,l\right)g\left(z_{\theta (l)}|x,l\right)}{\kappa (z_{\theta (l)})} \end{array}$$

κ(·) is the clutter intensity, $p_{D}\left(\cdot \right)$ and $q_{D}\left(\cdot \right)$ are respectively the detection and miss-detection probabilities, g(z|x,l) is the likelihood that (x,l) generates measurement *z*, θ:L→{0:|Z|} is a positive 1-1 map, and Θ is the entire set of such mappings.

For image observation, with the assumption that object template T(·) is not overlapped, i.e., $T\left(x_{1}\right)\neq T\left(x_{2}\right)$ given $x_{1}\neq x_{2}$, the separable likelihood is given by the following [26]:

(9) $$\begin{array}{cc} g(y|X) & =f_{B}\left(y\right)\prod_{x\in X}g_{y}\left(x\right) \end{array}$$

where *y* denotes the observed image, $f_{B}$ denotes the likelihood of the entire set of X, and $g_{y}\left(x\right)$ denotes the likelihood of a single object in the observed image. The designs of $f_{B}$ and $g_{y}$ vary according to the applications, characteristics of observed image, and object appearances.

First introduced in Reference [27], the concept of a hybrid TBD observation model takes advantage of both standard and separable likelihood models. Intuitively, while detected objects can be updated by the associated point measurements, the miss-detected objects can be updated directly from the image observation. This intuition can be described mathematically by defining the following:

(10) $$\begin{array}{cc} \sigma_{T}\left(T\left(y\right)|x,l\right) & ≜\frac{g_{T}\left(T\left(y\right)|x,l\right)}{g_{T}\left(T\left(y\right)|\emptyset \right)} \end{array}$$

The hybrid likelihood can then be written as follows [27]:

(11) $$\begin{array}{cc} g(y|X) & \propto \sum_{\theta \in \Theta (L(X))}\prod_{(x,l)\in X}\varphi_{y}^{\left(\theta (l)\right)}\left(x,l\right) \end{array}$$

where

$$\begin{array}{cc} \varphi_{y}^{\left(\theta (l)\right)}\left(x,l\right) & =\psi^{\left(\theta (l)\right)}\left(x,l|Z\right){\left[\sigma_{T}\left(T\left(y\right)|x,l\right)\right]}^{\delta_{0}\theta \left(l\right)} \end{array}$$

### 2.4. The Single Object RTS Smoother

Given a set single object observation $\left\{z_{1:N}\right\}$, where N≤K with *K* is the total number of tracking time steps, the smoothed density of an object state at time k≤N, $p(x_{k}|z_{1:N})$, is obtained as follows [36].

Initially, let the joint distribution of $x_{k}$ and $x_{k+1}$ be rewritten as follows:

(12) $$p\left(x_{k},x_{k+1}|z_{1:k}\right)=p\left(x_{k+1}|x_{k}\right)p\left(x_{k}|z_{1:k}\right)$$

Then, the distribution of $x_{k}$ given $x_{k+1}$ and $z_{1:k}$ is given as follows:

(13) $$p\left(x_{k}|x_{k+1},z_{1:k}\right)=\frac{p(x_{k},x_{k+1}|z_{1:k})}{p(x_{k+1}|z_{1:k})}$$

where $p\left(x_{k+1}|z_{1:k}\right)=\int p\left(x_{k+1}|x_{k}\right)p\left(x_{k}|z_{1:k}\right)dx_{k}$

From the Markov state-space model, we have the following property: $p\left(x_{k}|x_{k+1},z_{1:N}\right)=p\left(x_{k}|x_{k+1},z_{1:k}\right)$. Hence, we have the following:

(14) $$p\left(x_{k}|x_{k+1},z_{1:N}\right)=\frac{p(x_{k},x_{k+1}|z_{1:k})}{p(x_{k+1}|z_{1:k})}$$

Then, the joint distribution of $x_{k}$ and $x_{k+1}$ given the measurements set $z_{1:N}$ is given as follows:

(15) $$p\left(x_{k},x_{k+1}|z_{1:N}\right)=p\left(x_{k}|x_{k+1},z_{1:N}\right)p\left(x_{k+1}|z_{1:N}\right)$$

Finally, the smoothed density of state $x_{k}$ can then be obtained via the marginalization step as follows:

(16) $$p\left(x_{k}|z_{1:N}\right)=\int p\left(x_{k}|x_{k+1},z_{1:N}\right)p\left(x_{k+1}|z_{1:N}\right)dx_{k+1}$$

## 3. The Proposed Tracker

### 3.1. The Filtering Stage

For this tracker, we assume Gaussian distribution for the dynamic state of each object. At this first stage, the tracker carries out a standard multi-object filtering process to obtain the forward estimated labels and the measurements to label association history. In this subsection, we provide the forward filtering steps for both the GLMB filter (with standard measurements and hybrid measurements observations) and GLMB filter with object spawning.

#### 3.1.1. GLMB Filter without Objects Spawning

The procedure to estimate the state of a set of objects with the standard GLMB filter without including the spawning model in the transition kernel is given as follows.

Given a GLMB prior [16]

(17) $$\begin{array}{cc} \pi (X) & =\Delta \left(X\right)\sum_{(I,\xi )\in F(L)\times \Xi }\omega^{\left(I,\xi \right)}\delta_{I}\left(L\left(X\right)\right){\left[p^{\left(\xi \right)}\right]}^{X} \end{array}$$

and the standard observation model as in Equation (8), the filtering density in the next time step is given by the following [16]:

(18) $$\begin{array}{cc} \pi_{Z_{+}}\left(X\right) & \propto \Delta \left(X\right)\sum_{I,\xi ,I_{+},\theta_{+}}\omega^{\left(I,\xi \right)}\omega_{Z_{+}}^{\left(I,\xi ,I_{+},\theta_{+}\right)}\delta_{I_{+}}\left(L\left(X\right)\right){\left[p_{Z_{+}}^{\left(\xi ,\theta_{+}\right)}\right]}^{X} \end{array}$$

where $I\in F\left(L\right),\;\xi \in \Xi ,\;I_{+}\in F\left(L_{+}\right),\;\theta_{+}\in \Theta_{+}$ where ξ is the tracks to measurement association history and Ξ is the entire space of ξ.

$$\begin{array}{cc} \omega_{Z_{+}}^{\left(I,\xi ,I_{+},\theta_{+}\right)} & =1_{\Theta_{+}\left(I_{+}\right)}\left(\theta_{+}\right){\left[1-{\bar{P}}_{S}^{\left(\xi \right)}\right]}^{I-I_{+}}{\left[{\bar{P}}_{S}^{\left(\xi \right)}\right]}^{I⋂I_{+}}{\left[1-r_{B+}\right]}^{B_{+}-I_{+}}{\left[r_{B+}\right]}^{B_{+}\cap I_{+}}{\left[{\bar{\psi }}_{Z_{+}}^{\left(\xi ,\theta_{+}\right)}\right]}^{I_{+}} \end{array}$$

$$\begin{array}{cc} {\bar{P}}_{S}^{\left(\xi \right)}\left(l\right) & =\langle p^{\left(\xi \right)}\left(\cdot ,l\right),p_{S}\left(\cdot ,l\right)\rangle \end{array}$$

$$\begin{array}{cc} {\bar{\psi }}_{Z_{+}}^{\left(\xi ,\theta_{+}\right)}\left(l_{+}\right) & =\langle {\bar{p}}_{+}^{\left(\xi \right)}\left(\cdot ,l_{+}\right),\psi_{Z_{+}}^{\left(\theta_{+}\left(l_{+}\right)\right)}\left(\cdot ,l_{+}\right)\rangle \end{array}$$

$$\begin{array}{cc} p_{Z_{+}}^{\left(\xi ,\theta_{+}\right)}\left(x_{+},l_{+}\right) & =\frac{{\bar{p}}_{+}^{\left(\xi \right)}\left(x_{+},l_{+}\right)\psi_{Z_{+}}^{\left(\theta_{+}\left(l_{+}\right)\right)}\left(x_{+},l_{+}\right)}{{\bar{\psi }}_{Z_{+}}^{\left(\xi ,\theta_{+}\right)}\left(l_{+}\right)} \end{array}$$

$$\begin{array}{cc} {\bar{p}}_{+}^{\left(\xi \right)}\left(x_{+},l_{+}\right) & =1_{L}\left(\left\{l_{+}\right\}\right)\frac{\langle p_{S}\left(\cdot ,l_{+}\right)f_{S+}\left(x_{+}|\cdot ,l_{+}\right),p^{\left(\xi \right)}\left(\cdot ,l_{+}\right)\rangle }{{\bar{P}}_{S}^{\left(\xi \right)}\left(l_{+}\right)}+1_{B_{+}}\left(\left\{l_{+}\right\}\right)p_{B+}\left(x_{+},l_{+}\right) \end{array}$$

In tracking scenarios where raw spatial detection are also available, the hybrid model in Equation (11) can be used to replace the standard observation model with the probability of miss-detection being scaled by the spatial observation likelihood, i.e., given the GLMB prior as in Equation (17). The filtering density is then given as follows [27]:

(19) $$\begin{array}{cc} \pi_{y_{+}}\left(X\right) & \propto \Delta \left(X\right)\sum_{I,\xi ,I_{+},\theta_{+}}\omega^{\left(I,\xi \right)}\omega_{y_{+}}^{\left(I,\xi ,I_{+},\theta_{+}\right)}\delta_{I_{+}}\left(L\left(X\right)\right){\left[p_{y_{+}}^{\left(\xi ,\theta_{+}\right)}\right]}^{X} \end{array}$$

where

$$\begin{array}{cc} \omega_{y_{+}}^{\left(I,\xi ,I_{+},\theta_{+}\right)} & =1_{\Theta_{+}\left(I_{+}\right)}\left(\theta_{+}\right){\left[1-{\bar{P}}_{S}^{\left(\xi \right)}\right]}^{I-I_{+}}{\left[{\bar{P}}_{S}^{\left(\xi \right)}\right]}^{I⋂I_{+}}{\left[1-r_{B+}\right]}^{B_{+}-I_{+}}{\left[r_{B+}\right]}^{B_{+}\cap I_{+}}{\left[{\bar{\varphi }}_{y_{+}}^{\left(\xi ,\theta_{+}\right)}\right]}^{I_{+}} \end{array}$$

$$\begin{array}{cc} {\bar{\varphi }}_{y_{+}}^{\left(\xi ,\theta_{+}\right)}\left(l_{+}\right) & =\langle {\bar{p}}_{+}^{\left(\xi \right)}\left(\cdot ,l_{+}\right),\varphi_{y_{+}}^{\left(\theta_{+}\left(l_{+}\right)\right)}\left(\cdot ,l_{+}\right)\rangle \end{array}$$

$$\begin{array}{cc} p_{y_{+}}^{\left(\xi ,\theta_{+}\right)}\left(x_{+},l_{+}\right) & =\frac{{\bar{p}}_{+}^{\left(\xi \right)}\left(x_{+},l_{+}\right)\varphi_{y_{+}}^{\left(\theta_{+}\left(l_{+}\right)\right)}\left(x_{+},l_{+}\right)}{{\bar{\varphi }}_{y_{+}}^{\left(\xi ,\theta_{+}\right)}\left(l_{+}\right)} \end{array}$$

#### 3.1.2. GLMB Filter with Objects Spawning

For the prior density which is a GLMB density as in Equation (17) and the transition kernel defined in Equation (6), by applying the joint predict–update approach, a proposal density can be written as follows [30]:

(20) $$\begin{array}{cc} {\tilde{\pi }}_{+}\left(X_{+}|Z_{+}\right) & \propto \Delta \left(X_{+}\right)\sum_{I,\xi ,I_{+},\theta_{+}}\omega^{\left(I,\xi \right)}{\tilde{\omega }}_{Z_{+}}^{\left(I,\xi ,I_{+},\theta_{+}\right)}\delta_{I_{+}}\left(L\left(X_{+}\right)\right){\left[{\tilde{p}}_{Z_{+}}^{\left(\xi ,\theta_{+}\right)}\right]}^{X_{+}} \end{array}$$

$$\begin{array}{cc} {\tilde{\omega }}_{Z_{+}}^{\left(I,\xi ,I_{+},\theta_{+}\right)} & ={\left[r_{B+}\right]}^{B_{+}\cap I_{+}}{\left[1-r_{B+}\right]}^{B_{+}-I_{+}}{\left[{\bar{p}}_{S}\right]}^{I\cap I_{+}}{\left[1-{\bar{p}}_{S}\right]}^{I-I_{+}}{\left[{\bar{p}}_{T}\right]}^{S_{+}\cap I_{+}}{\left[1-{\bar{p}}_{T}\right]}^{S_{+}-I_{+}}, \end{array}$$

$$\begin{array}{cc} {\tilde{p}}_{Z_{+}}^{\left(\xi ,\theta_{+}\right)}\left(x_{+},l_{+}\right) & =\frac{{\tilde{p}}_{+}^{\left(\xi \right)}\left(x_{+},l_{+}\right)\psi_{Z_{+}}^{\left(\theta_{+}\left(l_{+}\right)\right)}\left(x_{+},l_{+}\right)}{{\tilde{\psi }}_{Z_{+}}^{\left(\xi ,\theta_{+}\right)}\left(l_{+}\right)}, \end{array}$$

$$\begin{array}{cc} {\tilde{p}}_{+}^{\left(\xi \right)}\left(x_{+},l_{+}\right) & =1_{B_{+}}\left(\left\{l_{+}\right\}\right)p_{B+}\left(x_{+},l_{+}\right)+1_{L}\left(\left\{l_{+}\right\}\right){\tilde{p}}_{S}^{\left(\xi \right)}\left(x_{+},l_{+}\right)+1_{S}\left(\left\{l_{+}\right\}\right){\tilde{p}}_{T}^{\left(\xi \right)}\left(x_{+},l_{+}\right), \end{array}$$

$$\begin{array}{cc} {\tilde{p}}_{S}^{\left(\xi \right)} & =\frac{\langle p_{S}\left(\cdot ,l_{+}\right)f_{S+}\left(x_{+}|\cdot ,l\right),p^{\left(\xi \right)}\left(\cdot ,l_{+}\right)\rangle }{{\bar{p}}_{S}^{\left(\xi \right)}\left(l_{+}\right)}, \end{array}$$

$$\begin{array}{cc} {\tilde{p}}_{T}^{\left(\xi \right)} & =\frac{\langle p_{T}\left(l_{+}\right)f_{T+}\left(x_{+}|\cdot ,l\right),p^{\left(\xi \right)}\left(\cdot ,l\right)\rangle }{{\bar{p}}_{T}^{\left(\xi \right)}\left(l_{+}\right)} \end{array}$$

$$\begin{array}{cc} {\bar{p}}_{S}^{\left(\xi \right)} & =\langle p^{\left(\xi \right)}\left(\cdot ,l\right),p_{S}\left(l_{+}\right)\rangle , \end{array}$$

$$\begin{array}{cc} {\bar{p}}_{T}^{\left(\xi \right)} & =\langle p^{\left(\xi \right)}\left(\cdot ,l\right),p_{T}\left(l_{+}\right)\rangle , \end{array}$$

$$\begin{array}{cc} {\tilde{\psi }}_{Z_{+}}^{\left(\xi ,\theta_{+}\right)}\left(l_{+}\right) & =\langle {\tilde{p}}_{+}^{\left(\xi \right)}\left(\cdot ,l_{+}\right),\psi_{Z_{+}}^{\left(\theta_{+}\left(l_{+}\right)\right)}\left(\cdot ,l_{+}\right)\rangle . \end{array}$$

From this proposal density, Gibbs’ sampler is applied to select high weight hypotheses. These hypotheses are subsequently used to form a standard GLMB density [30]:

(21) $$\hat{\pi }\left(X_{+}|Z_{+}\right)=\Delta \left(X_{+}\right)\sum_{I,\xi ,I_{+},\theta_{+}}\delta_{I_{+}}\left(L\left(X_{+}\right)\right){\hat{\omega }}_{+}^{\left(I,\xi ,I_{+},\theta_{+}\right)}\left(Z_{+}\right){\left[p_{B+}\psi_{+}^{\left(\theta_{+}\right)}\left(\cdot |Z_{+}\right)\right]}^{X_{B+}}\frac{{\left[{\hat{p}}_{+}^{\left(I,\xi ,I_{+},\theta_{+}\right)}\left(\cdot |Z_{+}\right)\right]}^{X_{S+}\cup X_{T+}}}{{\left[{\bar{p}}_{+}^{\left(I,\xi ,I_{+},\theta_{+}\right)}\left(\cdot |Z_{+}\right)\right]}^{I_{+}}}$$

$${\hat{\omega }}_{+}^{\left(I,\xi ,I_{+},\theta_{+}\right)}\left(Z_{+}\right)=\frac{\omega_{+}^{\left(I,\xi \right)}\left(I_{+}\right){\left[{\bar{p}}_{+}^{\left(I,\xi ,I_{+},\theta_{+}\right)}\left(\cdot |Z_{+}\right)\right]}^{I_{+}}}{\sum_{I,\xi ,I_{+},\theta_{+}}\omega_{+}^{\left(I,\xi \right)}\left(I_{+}\right){\left[{\bar{p}}_{+}^{\left(I,\xi ,I_{+},\theta_{+}\right)}\left(\cdot |Z_{+}\right)\right]}^{I_{+}}}$$

$$\begin{array}{cc} {\hat{p}}^{\left(I,\xi ,I_{+},\theta_{+}\right)}\left(x_{+},l_{+}|Z_{+}\right) & ≜1_{I_{+}}\left(\left\{l_{+}\right\}\right)\int p_{+}^{\left(I,\xi ,\theta_{+}\right)}\left(\left\{\left(x_{+},l_{+}\right),\left(x_{1,+},l_{1,+}\right),\ldots ,\left(x_{n,+},l_{n,+}\right)\right\}|Z_{+}\right)d\left(x_{1,+},\ldots ,x_{n,+}\right) \end{array}$$

$$\begin{array}{cc} {\bar{p}}^{\left(I,\xi ,I_{+},\theta_{+}\right)}\left(x_{+},l_{+}|Z_{+}\right) & ≜1_{B_{+}}\left(\left\{l_{+}\right\}\right)\langle p_{B+}\left(\cdot ,l_{+}\right),\psi_{+}^{\left(\theta_{+}\right)}\left(\cdot |Z_{+}\right)\rangle +\left(1-1_{B_{+}}\left(\left\{l_{+}\right\}\right)\right)\langle {\hat{p}}_{Z_{+}}^{\left(I,\xi ,I_{+},\theta_{+}\right)}\left(x_{+},l_{+}\right),1\rangle \end{array}$$

### 3.2. GLMB Multi-Scan Estimator

The concept of a multi-scan estimator is introduced in Reference [50]. Given a multi-scan GLMB from time step *j* to *k*, the cardinality distribution of the number of trajectories is given as follows:

(22) $$\mathrm{Pr}(|L\left(X_{j:k}\right)|=n)=\sum_{\xi ,I_{j:k}}\delta_{n}\left[\right|I_{j:k}\left|\right]w_{j:k}^{\left(\xi \right)}\left(I_{j:k}\right)$$

One possible form of a multi-scan estimator is to determine the component with the highest weight $w_{j:k}^{\left(\xi \right)}\left(I_{j:k}\right)$ given that it has the most probable cardinality by maximizing Equation (22). The expected trajectory estimate can then be computed from $p_{j:k}^{\left(\xi \right)}\left(\cdot ,l\right)$ for each $l\in I_{j:k}$.

In this work, we proposed modifications to the multi-scan estimator in Reference [50], which can eliminate track fragmentation and improve localization performance. The set of all estimated trajectories is updated at each time step via the most significant hypothesis with the most probable cardinality in the GLMB density. At the time step when state estimation is required, the information of estimated trajectories is passed into the estimator. At this stage, trajectories pruning is applied to eliminate short-term tracks. Subsequently, standard filtering and RTS smoothing techniques are applied on each trajectory to produce smoothed state estimates. The significance of this estimator is that it allows the application of smoothing techniques to improve the tracking accuracy while completely eliminates track fragmentation as the entire trajectory is estimated as a whole. In addition, as the complexity of the estimator depends only on the number of estimated tracks, the additional computational effort of the estimator is negligible compared to GLMB filtering. The detailed implementation of the estimator is given as in following subsections.

#### 3.2.1. Estimating the Trajectories

Given the GLMB density at the end of each filtering cycle, the GLMB filter estimate is the result of the maximum posteriori estimate of the cardinality with the means of the object states being conditioned on the estimated cardinality [15]. Given that the possible highest number of tracked objects is $N_{max}$, the cardinality distribution of the the objects set over a finite set of hypotheses ${\left\{{\left(I,\xi \right)}_{h}\right\}}_{h=1:H}$ is written as follows:

(23) $$\begin{array}{cc} {\rho \left(n\right)|}_{n=0:N_{max}} & =\sum_{H\in {\left\{{\left(I,\xi \right)}_{h}\right\}}_{h=1:H}}\omega^{\left(H\right)}\delta_{n}\left(\right|I^{\left(H\right)}\left|\right) \end{array}$$

The estimated cardinality is given as follows:

(24) $$\begin{array}{cc} \hat{N} & =argmax(\rho ) \end{array}$$

The estimated hypothesis is as follows:

(25) $$\begin{array}{cc} \hat{H} & =argmax_{\left(H\right)}\omega^{\left(H\right)}\delta_{\hat{N}}\left(\right|I^{\left(H\right)}\left|\right) \end{array}$$

The information from the filtering stage needs to be captured to facilitate the multi-scan estimator. At this stage, we represent a set of estimated trajectories at time *k* with a set of tuples defined as ${\hat{T}}_{k}≜\left\{\left({\hat{l}}_{1}^{k},{\hat{b}}_{{\hat{l}}_{1}^{k}},{\hat{\xi }}_{{\hat{l}}_{1}^{k}}\right),\ldots ,\left({\hat{l}}_{\hat{N}}^{k},{\hat{b}}_{{\hat{l}}_{\hat{N}}^{k}},{\hat{\xi }}_{{\hat{l}}_{\hat{N}}^{k}}\right)\right\}$, where ${\hat{l}}_{\hat{n}}^{k}$ is the label of estimated trajectory *n* at time *k*, ${\hat{b}}_{{\hat{l}}_{\hat{n}}^{k}}$ is its corresponding initial birth state (including the time of birth and initial kinematic state), and ${\hat{\xi }}_{{\hat{l}}_{\hat{n}}^{k}}$ is the corresponding association history. In addition, we also have set of tuples for all estimated trajectories $\hat{T}$ from time step 1 to current time step *k*. This set of tuples is updated at the end of each filtering time step via updating the association history and initial birth state of existing trajectories and adding new tuples to the set if the trajectories are new. The procedure to update the tuples set is given in Algorithm 1.

**Algorithm 1** Updating trajectories tuples
**Input:**${\hat{T}}_{k}=\left\{\left({\hat{l}}_{1}^{k},{\hat{b}}_{{\hat{l}}_{1}^{k}},{\hat{\xi }}_{{\hat{l}}_{1}^{k}}\right),\ldots ,\left({\hat{l}}_{\hat{N}}^{k},{\hat{b}}_{{\hat{l}}_{\hat{N}}^{k}},{\hat{\xi }}_{{\hat{l}}_{\hat{N}}^{k}}\right)\right\}$, $\hat{T}=\left\{\left({\hat{l}}_{1},{\hat{b}}_{{\hat{l}}_{1}},{\hat{\xi }}_{{\hat{l}}_{1}}\right),\ldots ,\left({\hat{l}}_{N},{\hat{b}}_{{\hat{l}}_{N}},{\hat{\xi }}_{{\hat{l}}_{N}}\right)\right\}$**Output:** The updated trajectories tuples set $\hat{T}$ **for**n=1 to $\hat{N}$  **if** ${\hat{l}}_{n}^{k}\in \left\{{\hat{l}}_{1},\ldots ,{\hat{l}}_{N}\right\}$
    Replace the tuple of label ${\hat{l}}_{n}^{k}$ in $\hat{T}$ with $\left({\hat{l}}_{n}^{k},{\hat{b}}_{{\hat{l}}_{n}^{k}},{\hat{\xi }}_{{\hat{l}}_{n}^{k}}\right)$  **else**    $\hat{T}=\hat{T}\cup \left({\hat{l}}_{n}^{k},{\hat{b}}_{{\hat{l}}_{n}^{k}},{\hat{\xi }}_{{\hat{l}}_{n}^{k}}\right)$  **end**
**end**



#### 3.2.2. Trajectories Pruning

For a set of estimated trajectories tuples from filtering stage $\hat{T}=\left\{\left({\hat{l}}_{1},{\hat{b}}_{{\hat{l}}_{1}},{\hat{\xi }}_{{\hat{l}}_{1}}\right),\ldots ,\left({\hat{l}}_{N},{\hat{b}}_{{\hat{l}}_{N}},{\hat{\xi }}_{{\hat{l}}_{N}}\right)\right\}$ the lifetime of a trajectory with label ${\hat{l}}_{n}$ is the length of the corresponding association history, which is given as follows:

(26) $$\begin{array}{cc} \tau (l_{n}) & =f_{length}\left({\hat{\xi }}_{{\hat{l}}_{n}}\right) \end{array}$$

where $f_{length}\left(\cdot \right)$ is the function that determines the length of the vector in its argument. If the length of a track is shorter than the threshold value $\tau_{t}$, i.e., $\tau \left(l_{n}\right)<\tau_{t}$, this trajectory will be removed from the set of estimated trajectories.

#### 3.2.3. Numerical Implementation of Single-Object Smoother

For completeness, we outline here the detailed numerical implementation of the single-object RTS smoother for both linear and nonlinear dynamic models with Gaussian assumption on the distribution of the states.

Given a linear dynamic model of the form

$$\begin{array}{cc} x_{+} & =Fx+q,z=Hx+r \end{array}$$

where *x* is the system state, *F* is the linear transformation matrix, *H* is the linear observation matrix, *q* and *r* are respectively the process and observation Gaussian noise, and *z* is the current time step measurement, the backward smoothing step over an interval N≤K (where *K* is the total number of tracking time steps) can be implemented with the standard RTS Smoother [32]. The details of the RTS smoother is given in Algorithm 2, where the superscript *s* denotes the smoothed results.

**Algorithm 2** Single-object Rauch–Tung–Striebel (RTS) smoother
**Input:** The filtered mean and covariance ${\left\{x_{k},P_{k}\right\}}_{k=1:N}$, *F*, *Q***Output:** The smoothed mean and covariance ${\left\{x_{k}^{s},P_{k}^{s}\right\}}_{k=1:N}$ **Initialization:**$x_{N}^{s}=x_{N}$ and $P_{N}^{s}=P_{N}$**for**k=N−1 down to 1  ${\bar{x}}_{k+1}=Fx_{k}$
  ${\bar{P}}_{k+1}=FP_{k}F^{T}+Q$
  $D=P_{k+1}F{\left({\bar{P}}_{k+1}\right)}^{-1}$
  $x_{k}^{s}=x_{k}+D\left(x_{k+1}^{s}-{\bar{x}}_{k+1}\right)$
  $P_{k}^{s}=P_{k}+D\left(P_{k+1}^{s}-{\bar{P}}_{k+1}\right)D^{T}$

**end**



For a nonlinear dynamic model, the RTS smoother can also be applied via the unscented transformation [39]. Given the dynamic model

$$\begin{array}{cc} x_{+} & =f(x,q),y=h(x,r) \end{array}$$

where *f* is the nonlinear state transition function and *h* is the nonlinear observation function, other variables are interpreted the same as in the linear model; the smoothed results can be inferred via the Unscented RTS (URTS) smoother [36]. The smoothing procedure is presented in Algorithm 3, and the readers are referred to Reference [39] for the detailed implementation of the unscented transform. Compared to the Sequential Monte Carlo method, unscented transform is less computationally expensive as the number of sigma points to approximate a Gaussian distribution is much lower than the number of particles to represent the entire density.

#### 3.2.4. Forward Filtering-Backward Smoothing of Trajectories

In this step, by using the measurement association history, the initial birth information (the state and the time at birth) in the estimated trajectories tuples set, and the measurements set, we apply standard single-object filtering and backward RTS smoothing techniques to produce a set of smoothed distributions of the trajectories. In this work, spatial distributions of tracks are assumed to be Gaussian distributed; hence, the estimated spatial distribution of track labeled *l* at time *k* is represented by the mean $m_{k}^{l}$ and the covariance $P_{k}^{l}$. The details of the procedure to produce the tracks distributions are given in Algorithm 4. The SingleObjectPrediction and SingleObjectUpdate functions are chosen according to the dynamic model, which can be Kalman prediction and Kalman update or their nonlinear variances. The linearity of the system also determines the SingleObjectSmoothing function, which takes the form of either Algorithm 2 or Algorithm 3 to smooth each individual trajectory. The output of the algorithm is the smoothed spatial distributions of all estimated trajectories, which is $\{m_{k}^{{\hat{l}}_{n}}$, $P_{k}^{{\hat{l}}_{n}}{\}}_{k_{i}^{{\hat{l}}_{n}}:k_{e}^{{\hat{l}}_{n}}}$. From this set of distributions, the mean values can be extracted to be used as the estimated states of the trajectories.

**Algorithm 3** Single-object Unscented RTS (URTS) smoother
**Input:** The filtered mean and covariance ${\left\{x_{k},P_{k}\right\}}_{k=1:N}$, $f(x_{+}|x)$, *Q***Output:** The smoothed mean and covariance ${\left\{x_{k}^{s},P_{k}^{s}\right\}}_{k=1:N}$ **Initialization:**$x_{N}^{s}=x_{N}$ and $P_{N}^{s}=P_{N}$**for**k=N−1 down to 1  $\left\{W_{i-1}^{\left(m\right)},W_{i-1}^{\left(c\right)},\left[\tilde{X_{i}^{x}};\tilde{X_{i}^{q}}\right]\right\}$ = UnscentedTransform $\left(x_{k},P_{k},Q\right)$  ${\tilde{X_{i}}}_{+}=f\left(\tilde{X_{i}^{x}},\tilde{X_{i}^{q}}\right)$
  ${\bar{x}}_{k+1}=\sum_{i}W_{i-1}^{\left(m\right)}{\tilde{X_{i}}}_{+}$
  ${\bar{P}}_{k+1}=\sum_{i}W_{i-1}^{\left(c\right)}\left({\tilde{X_{i}}}_{+}-{\bar{x}}_{k+1}\right){\left({\tilde{X_{i}}}_{+}-{\bar{x}}_{k+1}\right)}^{T}$
  ${\bar{C}}_{k+1}=\sum_{i}W_{i-1}^{\left(c\right)}\left(\tilde{X_{i}^{x}}-x_{k}\right){\left({\tilde{X_{i}}}_{+}-{\bar{x}}_{k+1}\right)}^{T}$
  $D={\bar{C}}_{k+1}{\left({\bar{P}}_{k+1}\right)}^{-1}$
  $x_{k}^{s}=x_{k}+D\left(x_{k+1}^{s}-{\bar{x}}_{k+1}\right)$
  $P_{k}^{s}=P_{k}+D\left(P_{k+1}^{s}-{\bar{P}}_{k+1}\right)D^{T}$

**end**



While the advantages are mentioned previously, this estimator is also subjected to certain drawbacks in challenging tracking scenarios. First, depending on the nature of the problem, the user needs to set an appropriate pruning threshold $\tau_{l}$ to prevent the estimator from deleting correct trajectories, especially when track identity switching is severe. Second, as the estimator relies on the latest hypothesis to produce estimates, the more this hypothesis deviates from the truth, the more inaccurate the entire estimation. In addition, in the case that wrong new tracks keep appearing in the set of trajectory estimates, overestimating of the number of tracks is also possible. However, the benefit from track fragmentation reduction and improvement of tracking accuracy given negligible computational effort is much more than the risk of incorrectly estimating the number of tracks, and the following simulation results are a strong demonstration of the benefits of our proposed tracker.

**Algorithm 4** Trajectory forward filtering-backward smoothing
**Input:**$\hat{T}=\left\{\left({\hat{l}}_{1},{\hat{b}}_{{\hat{l}}_{1}},{\hat{\xi }}_{{\hat{l}}_{1}}\right),\ldots ,\left({\hat{l}}_{N},{\hat{b}}_{{\hat{l}}_{N}},{\hat{\xi }}_{{\hat{l}}_{N}}\right)\right\}$, $\left\{Z_{1},\ldots ,Z_{K}\right\}$**Output:** The estimated trajectories $\{\{m_{k}^{{\hat{l}}_{1}}$, $P_{k}^{{\hat{l}}_{1}}{\}}_{k_{i}^{{\hat{l}}_{1}}:k_{e}^{{\hat{l}}_{1}}},\ldots ,\{m_{k}^{{\hat{l}}_{N}}$, $P_{k}^{{\hat{l}}_{N}}{\}}_{k_{i}^{{\hat{l}}_{N}}:k_{e}^{{\hat{l}}_{N}}}\}$ **for**n=1 to *N*  Initialize $\left\{{\bar{m}}_{k_{i}^{{\hat{l}}_{n}}}^{l_{n}},{\bar{P}}_{k_{i}^{{\hat{l}}_{n}}}^{l_{n}}\right\}$ from the initial birth ${\hat{b}}_{{\hat{l}}_{n}}$  $\{{\tilde{m}}_{k_{i}^{{\hat{l}}_{n}}}^{l_{n}},{\tilde{P}}_{k_{i}^{{\hat{l}}_{n}}}^{l_{n}}\}=$ SingleObjectUpdate $\left({\bar{m}}_{k_{i}^{{\hat{l}}_{n}}}^{l_{n}},{\bar{P}}_{k_{i}^{{\hat{l}}_{n}}}^{l_{n}},z_{{\hat{\xi }}_{{\hat{l}}_{n}}^{k_{i}^{{\hat{l}}_{n}}}}^{k_{i}^{{\hat{l}}_{n}}}\right)$  **for** *k* from $k_{i}^{{\hat{l}}_{n}}+1$ to $k_{e}^{{\hat{l}}_{n}}$    $\{{\bar{m}}_{k}^{l_{n}},{\bar{P}}_{k}^{l_{n}}\}=$ SingleObjectPrediction $\left({\tilde{m}}_{k-1}^{l_{n}},{\tilde{P}}_{k-1}^{l_{n}}\right)$    **if** ${\hat{\xi }}_{{\hat{l}}_{n}}^{k}=0$
      $\left\{{\tilde{m}}_{k}^{l_{n}},{\tilde{P}}_{k}^{l_{n}}\right\}=\left\{{\bar{m}}_{k}^{l_{n}},{\bar{P}}_{k}^{l_{n}}\right\}$
    **else**      $\{{\tilde{m}}_{k}^{l_{n}},{\tilde{P}}_{k}^{l_{n}}\}=$ SingleObjectUpdate $\left({\bar{m}}_{k}^{l_{n}},{\bar{P}}_{k}^{l_{n}},z_{{\hat{\xi }}_{{\hat{l}}_{n}}^{k}}^{k}\right)$    **end**  **end**  $\{m_{k}^{{\hat{l}}_{n}}$, $P_{k}^{{\hat{l}}_{n}}{\}}_{k_{i}^{{\hat{l}}_{n}}:k_{e}^{{\hat{l}}_{n}}}=$ SingleObjectSmoothing $(\{{\tilde{m}}_{k}^{{\hat{l}}_{n}}$, ${\tilde{P}}_{k}^{{\hat{l}}_{n}}{\}}_{k_{i}^{{\hat{l}}_{n}}:k_{e}^{{\hat{l}}_{n}}})$
**end**



## 4. Experimental Results

### 4.1. Simulation Results

#### 4.1.1. Linear Dynamic Model

In this experiment, we use a constant velocity model for the dynamic of the system. The state vector consists of information regarding the planar position and the velocity of the objects, which is $x_{k}={\left[p_{x},p_{y},{\dot{p}}_{x},{\dot{p}}_{y}\right]}^{T}$; while the measurement vector contains the position of the object, which is $z_{k}={\left[z_{x},z_{y}\right]}^{T}$. The transition and observation models are given respectively as follows:

$$\begin{array}{cc} f_{+}\left(x_{+}|x\right) & =N(x_{+};Fx,Q) \end{array}$$

$$\begin{array}{cc} h(z|x) & =N(z;Hx,R) \end{array}$$

where $F=\left[\begin{array}{cc} I_{2} & \Delta I_{2}\\ 0_{2} & I_{2} \end{array}\right]$, $Q=\sigma_{v}^{2}\left[\begin{array}{cc} \frac{\Delta^{4}}{4}I_{2} & \frac{\Delta^{3}}{2}I_{2}\\ \frac{\Delta^{3}}{2}I_{2} & \Delta^{2}I_{2} \end{array}\right]$, $H=\left[\begin{array}{cc} I_{2} & 0_{2} \end{array}\right]$, $R=\sigma_{\epsilon }^{2}I_{2}$. Particularly, in this experiment, we set $\sigma_{v}=5$ m/s and $\sigma_{\epsilon }=15\;$ m.

**Figure 1:**
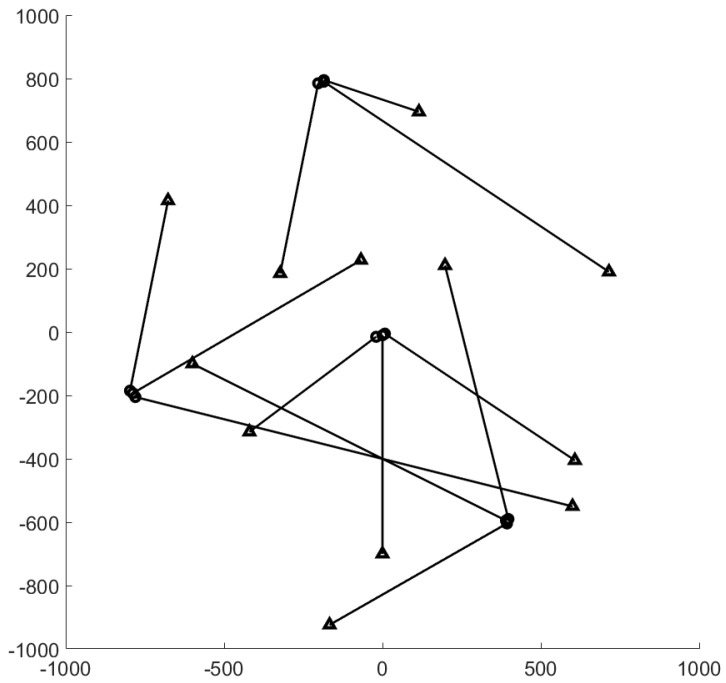
Ground truth for linear dynamic scenario (circle: track start position, triangle: track end position).

The surveillance region is the [−1000,1000]m×[−1000,1000]m area, the total time step is K=100, and Δ=1. The ground truth plot for this experiment is given in Figure 1. The surviving probability is set to $p_{S}=0.99$, and the detection probability is $p_{D}=0.95$. Clutter rate is set to 66 false alarms per scan. The birth probability is set to $r_{B}=0.03$. The states of expected births are $m_{B}^{\left(1\right)}={\left[0.1,0,0.1,0\right]}^{T}$, $m_{B}^{\left(2\right)}={\left[400,0,-600,0\right]}^{T}$, $m_{B}^{\left(3\right)}={\left[-800,0,-200,0\right]}^{T}$, and $m_{B}^{\left(4\right)}={\left[-200,0,800,0\right]}^{T}$. The covariance matrix at birth is $P_{B}=diag\left(\left[10,10,10,10\right]\right)$. The number of hypotheses for GLMB filter is capped at 20,000 components. In this experiment, we smooth the entire tracking interval from k=1 to k=K. The threshold for the smoother to prune the track is set to $\tau_{t}=3$ time steps.

**Figure 2:**
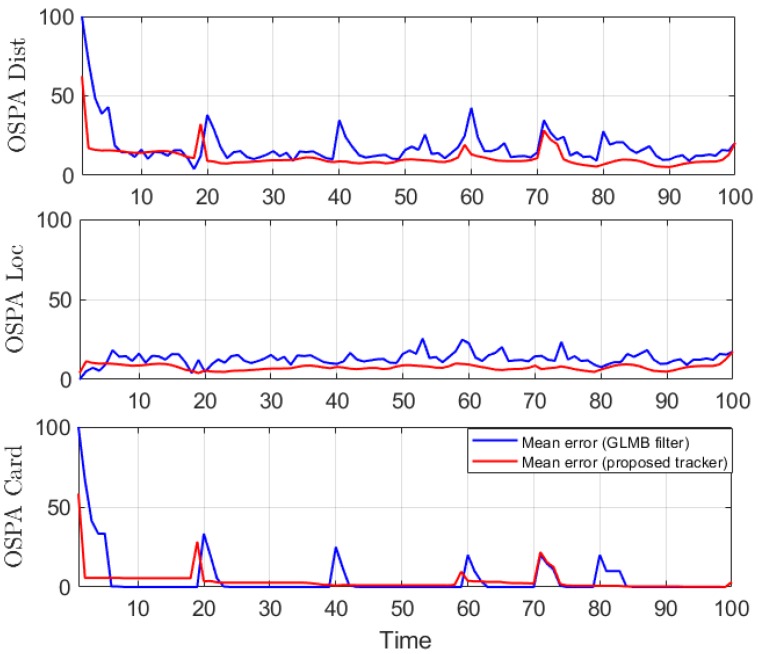
OSPA error for linear dynamic scenario.

**Figure 3:**
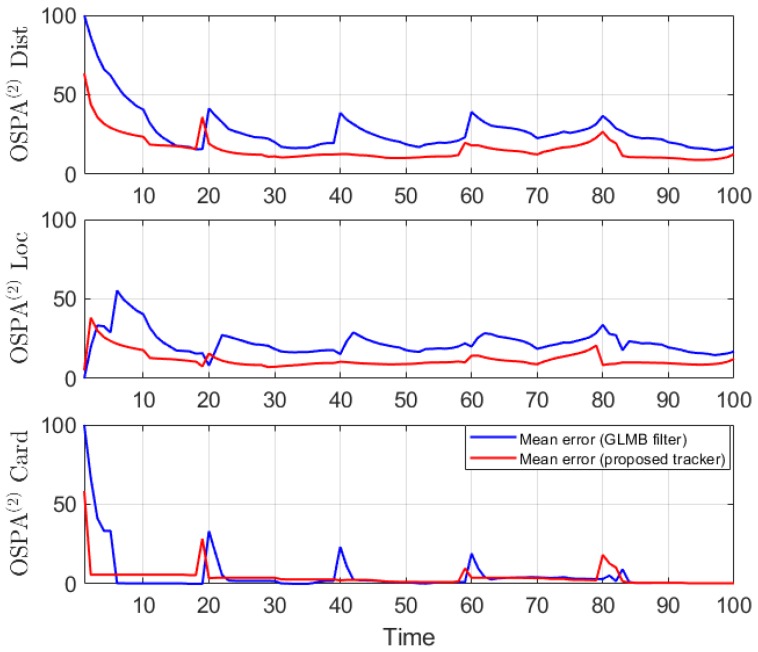
OSPA^2^ error for linear dynamic scenario.

**Figure 4:**
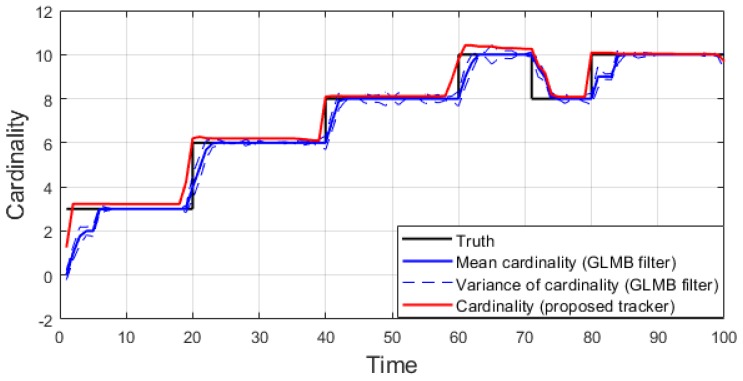
Estimated cardinality for linear dynamic scenario.

We conduct the experiment over 100 Monte Carlo runs. The means of the estimated Optimal Subpattern Assignment (OSPA) error [55] and OSPA^2^ error [52,56] are given respectively in Figure 2 and Figure 3. Figure 4 shows the GLMB filter and proposed tracker-estimated cardinality of objects set for each time step along with the true values.

#### 4.1.2. Nonlinear Dynamic Model

For the demonstration of the nonlinear tracking scenario, we use a constant turn model with 5-D state vector $x_{k}={\left[p_{x},p_{y},{\dot{p}}_{x},{\dot{p}}_{y},\omega \right]}^{T}$, where ω is the object’s turn rate. The transition density is given as follows:

$$\begin{array}{cc} f_{+}\left(x_{+}|x\right) & =N(x_{+};F\left(\omega \right)x,Q) \end{array}$$

where $F\left(\left[\begin{array}{c} p_{x}\\ \dot{p_{x}}\\ p_{y}\\ \dot{p_{y}}\\ \omega \end{array}\right]\right)=\left[\begin{array}{ccccc} 1 & \frac{\sin(\omega \Delta )}{\omega } & 0 & -\frac{1-\cos(\omega \Delta )}{\omega } & 0\\ 0 & \cos(\omega \Delta ) & 0 & -\sin(\omega \Delta ) & 0\\ 0 & \frac{1-\cos(\omega \Delta )}{\omega } & 1 & \frac{\sin(\omega \Delta )}{\omega } & 0\\ 0 & \sin(\omega \Delta ) & 0 & \cos(\omega \Delta ) & 0\\ 0 & 0 & 0 & 0 & 1 \end{array}\right]$, $Q_{\zeta =}\left[\begin{array}{cc} \sigma_{\omega }^{2}GG^{T} & 0\\ 0 & \sigma_{v}^{2} \end{array}\right]\;\mathrm{and}\;G=\left[\begin{array}{cc} \Delta^{2}/2 & 0\\ \Delta & 0\\ 0 & \Delta^{2}/2\\ 0 & \Delta \end{array}\right]$.

In this experiment, we set $\sigma_{\omega }=\pi /180\;$ rad/s and $\sigma_{v}=5\;$ m/s. The observation model is given as the bearing and range detection of the 2D vector $z_{k}={\left[\theta ,r\right]}^{T}$ with $\sigma_{\theta }=\pi /90\;$ rad and $\sigma_{r}=5\;$ m.

**Figure 5:**
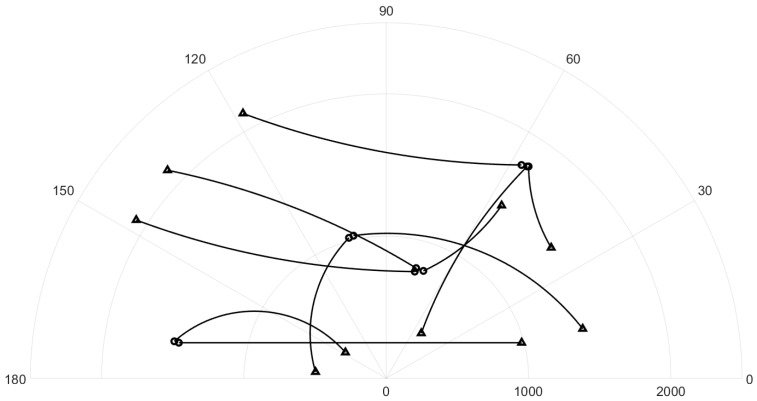
Ground truth for nonlinear dynamic scenario (circle: track start position, triangle: track end position).

The surveillance region is the half disc of the radius 2000 m with K=100 time steps and Δ=1. The ground truth for this experiment is given in Figure 5. The surviving probability is set to $p_{S}=0.99$ and the detection probability is $p_{D}=0.95$. Clutter rate is set to 66 false alarms per scan. The expected birth states are $m_{B}^{\left(1\right)}={\left[-1500,0,250,0,\pi /180\right]}^{T}$, $m_{B}^{\left(2\right)}={\left[-250,0,1000,0,\pi /180\right]}^{T}$, $m_{B}^{\left(3\right)}={\left[250,0,750,0,\pi /180\right]}^{T}$, and $m_{B}^{\left(4\right)}={\left[1000,0,1500,0,\pi /180\right]}^{T}$ with $r_{B}=0.02$, and the birth covariance is $P_{B}=diag\left(\left[50,50,50,50,\pi /30\right]\right)$. The number of hypotheses is also capped at 20,000 components. The smoothing interval is the entire tracking sequence from k=1 to k=K. We also set the track pruning threshold for the smoother to 3 time steps in this experiment.

**Figure 6:**
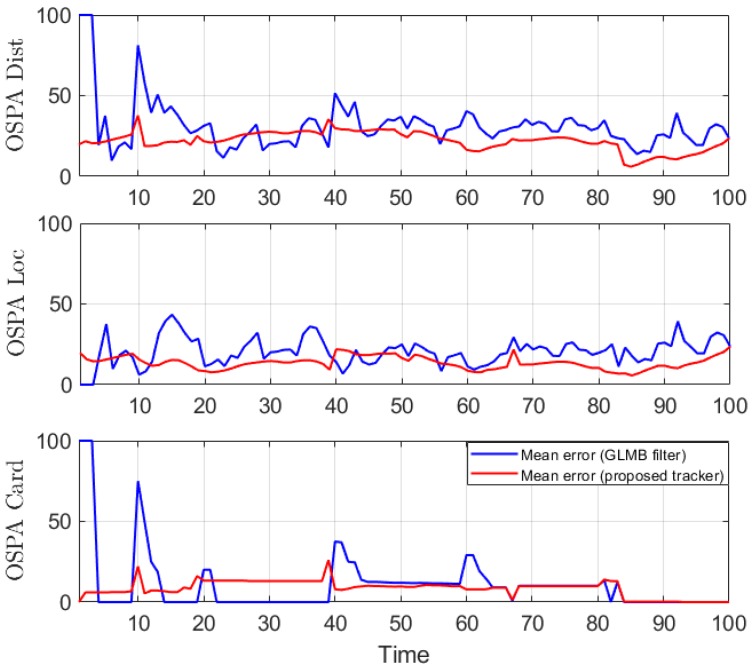
OSPA error for nonlinear dynamic scenario.

**Figure 7:**
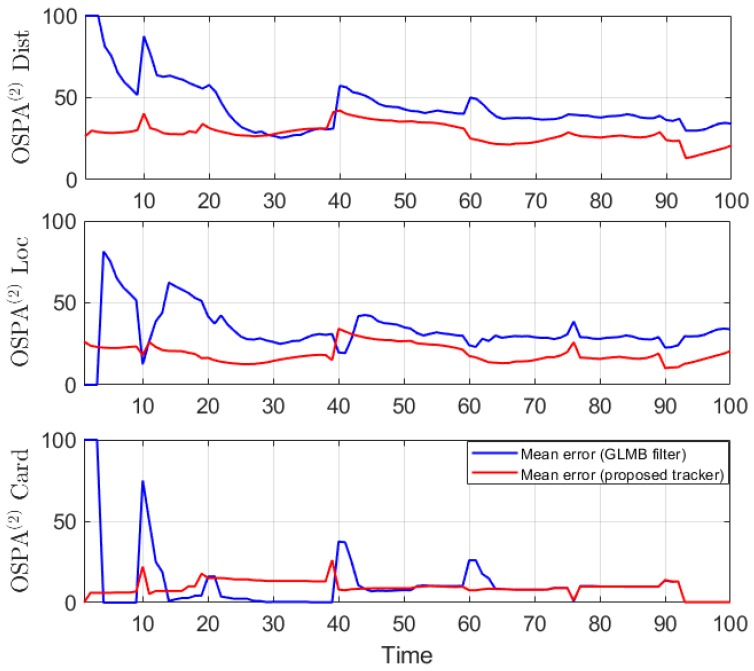
OSPA^2^ error for nonlinear dynamic scenario.

**Figure 8:**
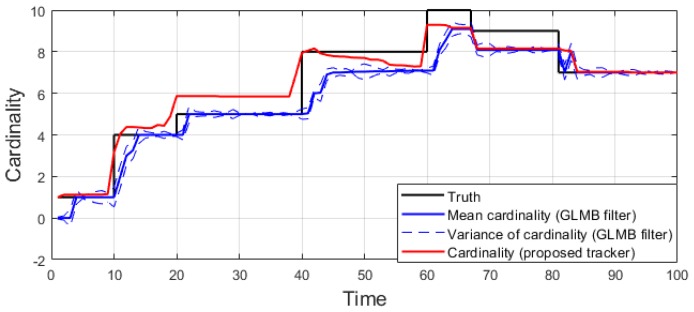
Estimated cardinality for nonlinear dynamic scenario.

For this scenario, we also test the performance of the tracker over 100 Monte Carlo runs. The means of OSPA error and OSPA^2^ error of the estimates are plotted in Figure 6 and Figure 7, respectively, while the set cardinality is shown in Figure 8.

#### 4.1.3. Hybrid TBD Observation Model

In this simulation, we use the hybrid measurement model to track objects following a linear dynamic motion model. The surveillance region is 100×100 pixels with image cell size of 1, total time step of K=100, and Δ=1. The observation are the raw images, which are arrays of pixels. In particular, for a pixel *i* at the image coordinate $\left(a^{\left(i\right)},b^{\left(i\right)}\right)$, the array value is given as follows [26,27]:

(27) $$\begin{array}{cc} y^{\left(i\right)} & =\left[\sum_{x\in X}\frac{I_{k}}{2\pi \sigma_{h}}\exp\left(-\frac{{\left(a^{\left(i\right)}-p_{x}\right)}^{2}+{\left(b^{\left(i\right)}-p_{y}\right)}^{2}}{2\sigma_{h}^{2}}\right)\right]+w^{\left(i\right)} \end{array}$$

where $w^{\left(i\right)}∽N\left(0,\sigma_{y}\right)$ is Gaussian noise. In this experiment, we set $\sigma_{h}=4$ and $\sigma_{y}=1$. We choose the value of $I_{k}$ such that the signal to noise ratio (SNR) varies over the range 7 to 10 dB. For the observation model from the perspective of the filter, we fix its SNR value to 10 dB. From the raw images, we then use hard-shareholding to extract the points measurements at each frame.

**Figure 9:**
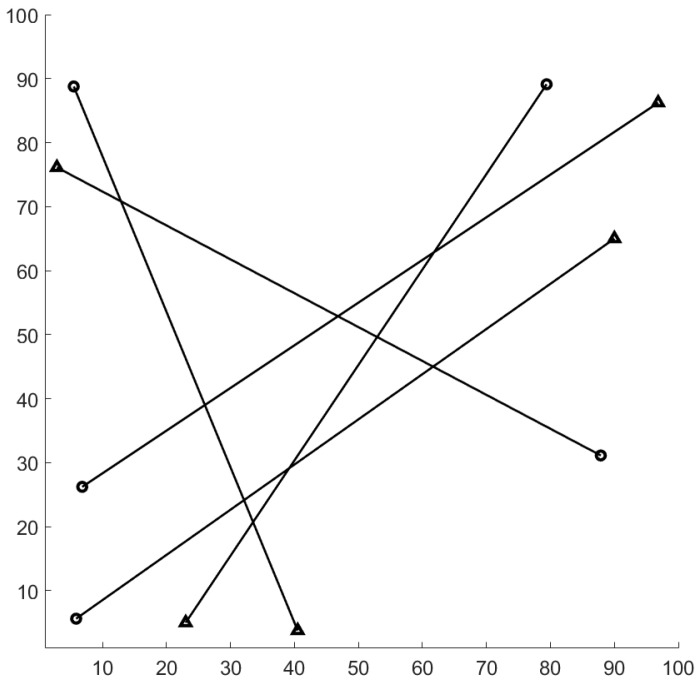
Ground truth for a hybrid track-before-detect (TBD) scenario (circle: track start position, triangle: track end position).

**Figure 10:**
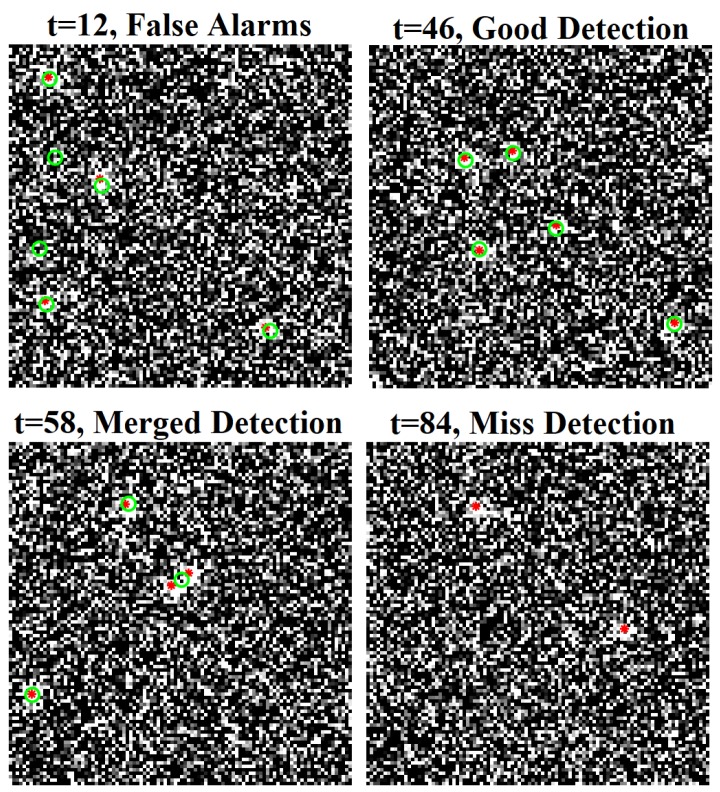
Samples of raw images and point observations for a hybrid TBD scenario (red asterisk: ground truth position, green circle: point detection).

The dynamic model and standard observation model are similar to the ones in Section 4.1.1 with $\sigma_{v}=1$ pixel/s, $p_{S}=0.98$, and $\sigma_{\epsilon }=4$ pixels with a clutter rate of 10. The expected new births states are $m_{B}^{\left(1\right)}={\left[5,0,25,0\right]}^{T}$, $m_{B}^{\left(2\right)}={\left[5,0,90,0\right]}^{T}$, $m_{B}^{\left(3\right)}={\left[80,0,90,0\right]}^{T}$, $m_{B}^{\left(4\right)}={\left[5,0,5,0\right]}^{T}$, and $m_{B}^{\left(5\right)}={\left[90,0,30,0\right]}^{T}$ with the covariance of $P_{B}=diag\left(\left[3,2,3,2\right]\right)$ and the probability $r_{B}$ of 0.03. The ground truth location of objects is shown in Figure 9 while Figure 10 shows samples of raw image observation along with points detection. The implementation of the filtering phase is as the same as in Reference [27]. The smoothing interval is set to the entire tracking time with the track pruning threshold of the smoother set to 3 time steps.

**Figure 11:**
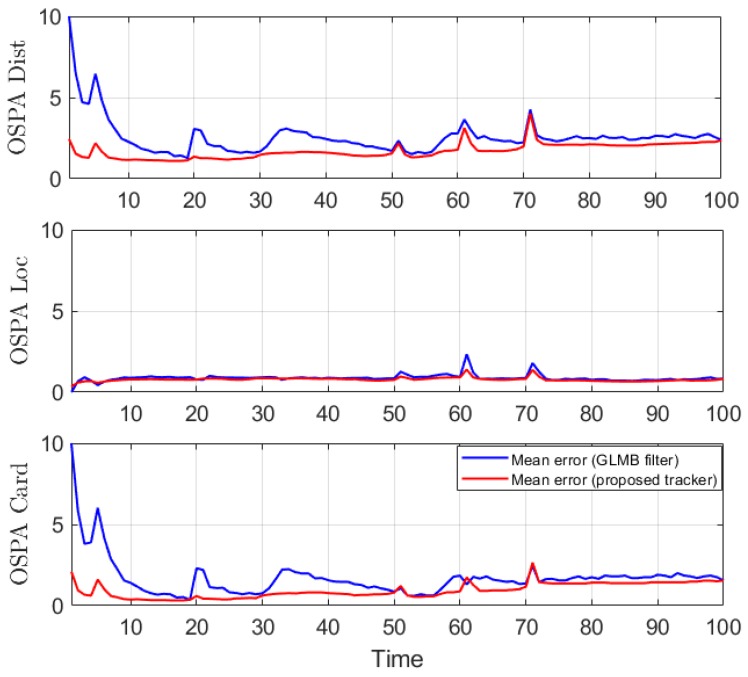
OSPA error for a hybrid TBD scenario.

**Figure 12:**
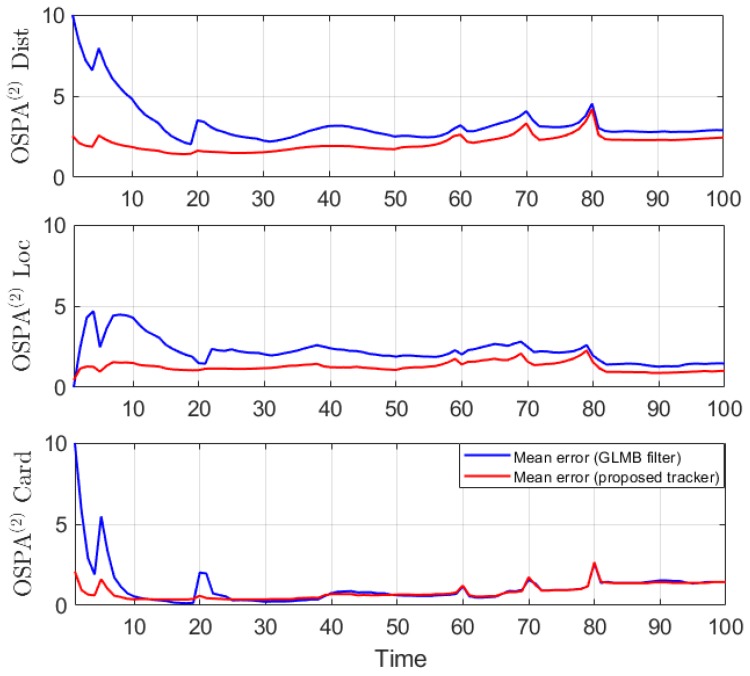
OSPA^2^ error for a hybrid TBD scenario.

**Figure 13:**
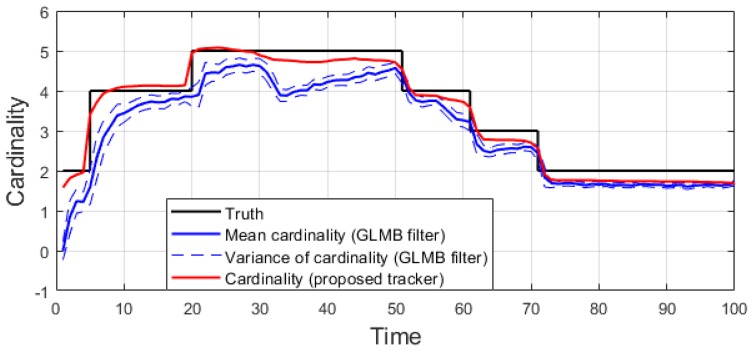
Estimated cardinality for a hybrid TBD scenario.

This experiment is run over 100 Monte Carlo trials. The means of OSPA error and OSPA^2^ error are shown respectively in Figure 11 and Figure 12. The estimated cardinality is plotted in Figure 13.

#### 4.1.4. Discussion on the Simulation Results

For all simulated experiments, we observe lower OSPA and OSPA^2^ errors for the proposed tracker compared to the GLMB filter results. In the first two experiments with the standard observation model, as the clutter rate is high, the filtered-only trajectories jiggle around the true paths due to false measurements. In Figure 2 and Figure 6 as well as in Figure 3 and Figure 7, the overall errors of the GLMB filter estimates are higher than of the proposed tracker estimates. The reduction of localization error contribute mainly to the improvement of the tracking performance. From the cardinality plots in Figure 4 and Figure 8, on average, the proposed tracker slightly improves estimate cardinality performance as it is able to eliminate track fragmentation while eliminating incorrect tracks at some time steps.

In the hybrid TBD tracking experiment, as tracks are miss-detected due to low SNR, the proposed tracker improves tracking performance by eliminating track fragmentation. Not much localization error is reduced by the smoother step as the GLMB filter produces relatively good tracking results. The OSPA and OSPA^2^ results presented in Figure 11 and Figure 12 show slight improvement of the proposed tracker results compared to GLMB filter tracking results. However, the cardinality plot in Figure 13 clearly indicates that the proposed tracker is able to improve the estimated cardinality between time step 30 and 40.

**Figure 14:**
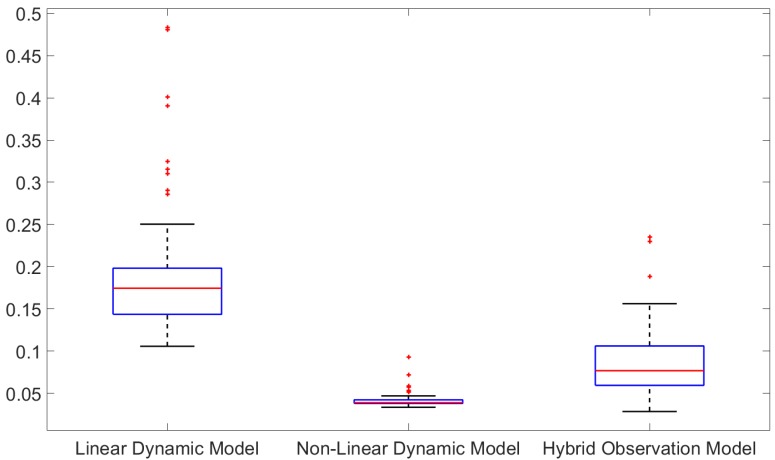
Percentage of smoothing time over filtering time.

The run time for all simulated scenario is given in Figure 14 in terms of the percentage of extra computational time of the proposed tracker over the computational time of the filtering step only. It is shown that the extra computational time is negligible in all three tracking scenarios with the extra computational time of the proposed tracker less than 0.5% of the filtering computational time. However, the main disadvantage is that the tracker needs to wait until the end of the smoothing interval to be able to produce tracking results.

### 4.2. Application to Cell Microscopy

**Figure 15:**
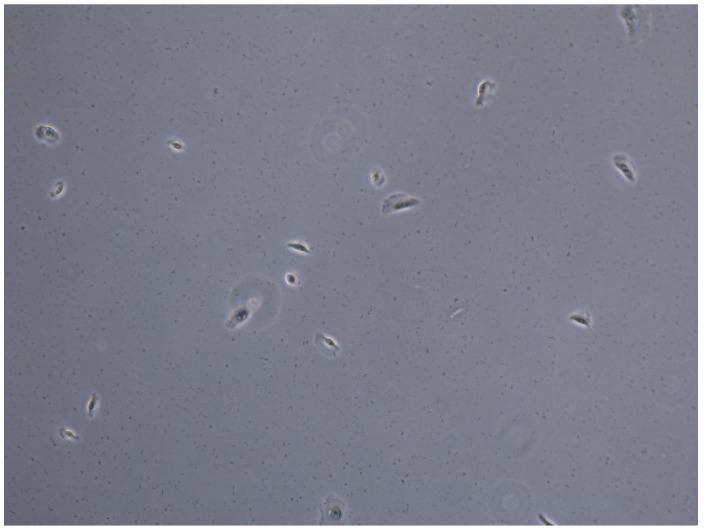
Snapshot of biological cell sequence.

In this experiment, we attempt to track biological cells from a sequence of images containing 90 frames by using the proposed tracker. A snapshot of the sequence is shown in Figure 15. In this application, we use the constant turn rate for the dynamic model as in Section 4.1.2 and the standard observation model as in Section 4.1.1. We also implement the measurement driven model as described in Reference [20]. For the first time step, the birth rate is set to a very high value (≈1) to initialize objects. Subsequently, the birth rate is capped at 10^−7^. The standard deviation of the turn rate noise is π/90 rad/s, and the standard deviation of the velocity noise is 5 pixels/frame. The number of hypotheses is capped at 10,000. The detection rate is set to 0.88, and the surviving rate and the spawning rate are 0.999 and 0.035, respectively. The clutter rate is set to 0.05. The cell spawning model is the same as described in Reference [57] with the covariance of the spawning model given as $Q_{T}=\left[\begin{array}{ccccc} 40 & 0 & 0 & 0 & 0\\ 0 & 5 & 0 & 0 & 0\\ 0 & 0 & 40 & 0 & 0\\ 0 & 0 & 0 & 5 & 0\\ 0 & 0 & 0 & 0 & \pi /90 \end{array}\right]$ and the smoothing interval set to the entire image sequence. In this application, we set the track pruning threshold of the estimator to 3 time steps.

**Figure 16:**
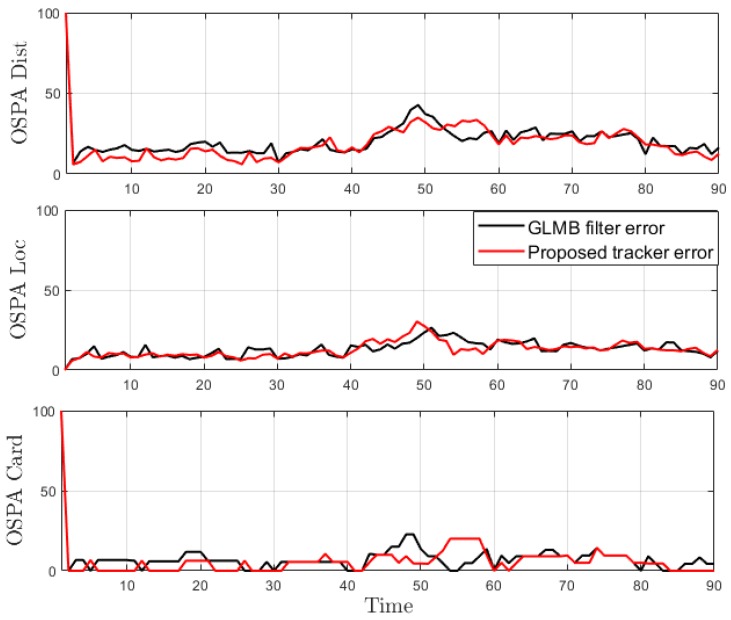
OSPA error for tracking biological cells.

**Figure 17:**
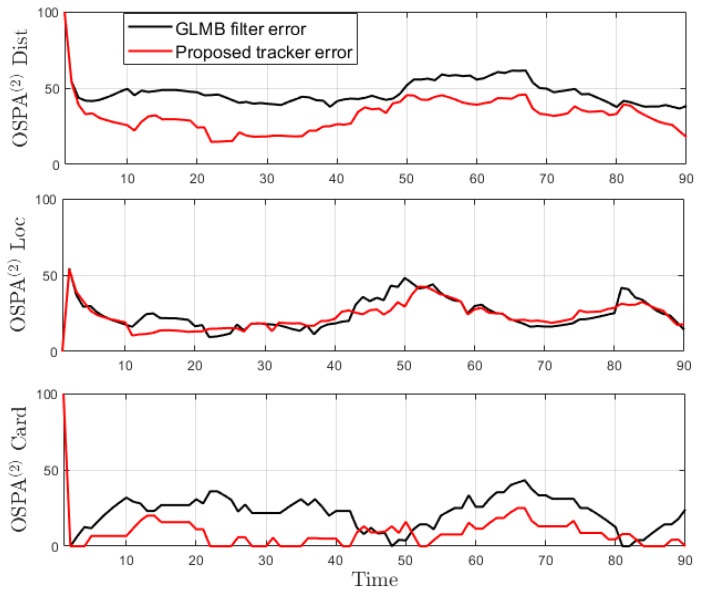
OSPA^2^ error for tracking biological cells.

**Figure 18:**
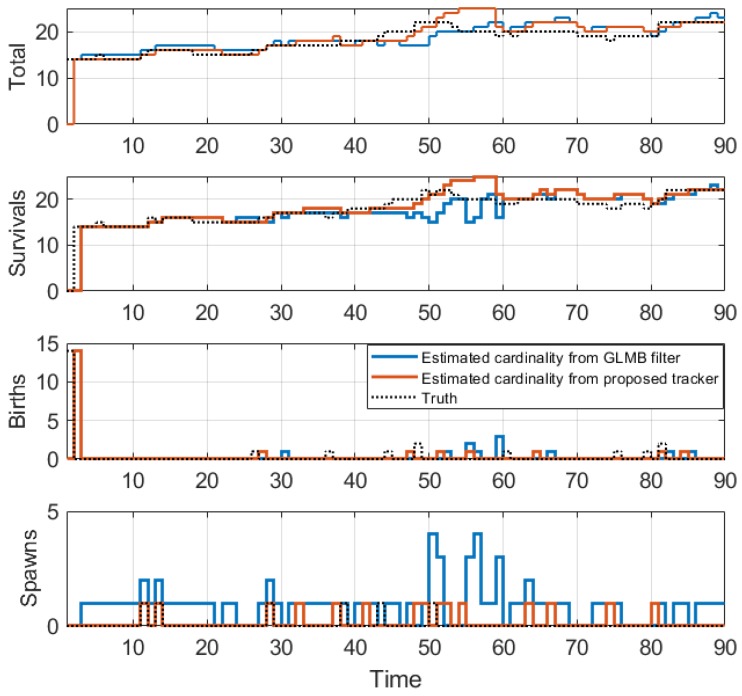
Estimated cardinality for tracking biological cells.

**Figure 19:**
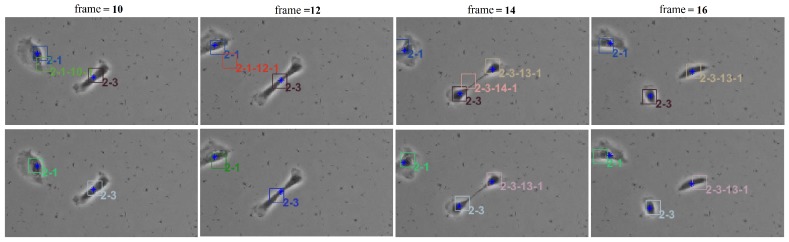
The tracked image sequences of biological cells with blue asterisks denoting points detection. Top row: Generalized Labeled Multi-Bernoulli (GLMB) filter tracking results. Bottom: Proposed tracker tracking results.

From the tracking results, significant improvement is observed as the proposed tracker is able to eliminate incorrect spawned tracks. While the OSPA error in Figure 16 shows similar performance for the GLMB filter and the proposed tracker, the improvement is clearly reflected in the OSPA^2^ cardinality error plots in Figure 17. From the cardinality plot in Figure 18, the estimated cardinality from our tracker is much closer to the true values as fewer incorrect spawned tracks are estimated. In this experiment, there is not much difference between the GLMB filter and the proposed tracker estimates localization error due to the mismatch between the dynamic model and actual motion of the cells. Finally, in Figure 19, we illustrate the improved tracking results in terms of tracking sequence for several time steps at a selected region where the cell splitting process occurs.

## 5. Conclusions

In this paper, we detailed the implementation of a new tracker based on GLMB filter and a modified multi-scan estimator. In addition to lowering the localization error by performing RTS smoother on each individual estimated trajectory, the proposed tracker can also reduce cardinality errors by deleting the short-term tracks via track management and by completely eliminating track fragmentation. The computation time is shown to contribute to less than 0.5% of the total tracking time, although a fixed delay time is needed before the tracker can produce the estimate. Therefore, in applications when real-time updates are not required, the proposed tracker can be used to improve the tracking results given negligible extra computation time. However, as the smoothing results strongly depend on the quality of the estimates obtained from the forward filtering step, if the filtered estimate experiences strong distortion, the performance of the proposed tracker degrades significantly.
